# Supplementary material for: Patterns of Intron Gain and Loss in Fungi
Source: PLoS Biol. 2004 Nov 30;2(12):e422. doi: 10.1371/journal.pbio.0020422 (PMC532390; doi:10.1371/journal.pbio.0020422)
Supplement: Table S1 — Also available at http://genes.mit.edu/NielsenEtAl/. (4.3 MB ZIP). [file pbio.0020422.st001.zip › NielsenEtAl/html/495.html]

AN0137.1.NCU01747.1.MG06704.1.FG01473.1


```
 CLUSTAL W (1.82) Multiple Sequence Alignments - Introns Inserted


Sequence 1: NCU01747.1	1245 aa
Sequence 2: FG01473.1	1173 aa
Sequence 3: MG06704.1	1303 aa
Sequence 4: AN0137.1	1205 aa
Alignment Length: 1371 aa
Number Identitical Residues: 555 aa
Alignment Score (without introns) 27017


MG06704.1 	MWASGVPRESRWEQDTRAEVELVGKRL1PQVPYGHAHSARPNTTGLLRFQAWLMHVARKP
NCU01747.1	---------------------------~--------------------------------
FG01473.1 	---------------------------~--------------------------------
AN0137.1  	---------------------------~--------------------------------
          	                                                            

MG06704.1 	ILPVGTRCCSKS1KGIPPFRPELIGIPGWLSMPTG1AYTDK0ANSGEPQQCTRLSVNNMK
NCU01747.1	------------~----------------------~-----~----------------MK
FG01473.1 	------------~----------------------~-----~----------------MK
AN0137.1  	------------~----------------------~-----~----------------MK
          	                                                          **

MG06704.1 	FGRN2LPRNQVPEWSSAYIDYKGLKKLIKAAGATSKNGGQADLAE1FFFALDRNLEDVDS
NCU01747.1	FGRN2LPRNQVPEWAAFYINYKGLKKLIKAAQGAAKNGEPVDLAE~FFFALDRNLEDVDS
FG01473.1 	FGRN2LPRNQVPEWAASYINYKGLKKLVKAAAEKAKNGEKVDPAE1FFFALDRNLEDVDF
AN0137.1  	FGRN~LPRNVVPEWSSSYIRYKALKKLIKSLADRVRAGHEADLAG1FFYSLDRNLEDVDH
          	**** **** ****:: ** **.****:*:     : *  .* *  **::********* 

MG06704.1 	FYNKKFADACRRLRLLHDRYGSSVDAISNLDQDEVEELMGAMLELRSKLRQLQWFGEINR
NCU01747.1	FYNKKYAEAYRRLKVLQDRYGKTPEIVSNLDDDEVEELMGALLELRSQFRKLQWFGEINR
FG01473.1 	FYNKKLAEFCRRLNLLQNRYGRTPDVVATLDQDEVEEVMGALLELRSQFRNLQWFGEINR
AN0137.1  	FYNKKYADFSRRLKLLSDRYAHNLDG-SHLDSDDVEDLLAALLELRGQFRKLQWYGEVNR
          	***** *:  ***.:* :**. . :  : **.*:**:::.*:****.::*:***:**:**

MG06704.1 	RGFVKITKKLDKKVP-GTVTQDRYIATRVDPCPFAKDNTIVRILNDINKWLSVLGDAQNM
NCU01747.1	RGFVKITKKLDKKVPNTKSTQHNYISTKVDLLPFAKDTAISLLLTEINKWLSKLGDAQNI
FG01473.1 	RGFVKITKKLDKKVP-DLVTQRPYITTKVDVKPFAKEANTARLLNEINRWMSVLSEAQTF
AN0137.1  	RGFNKITKKLDKKVG--AQAQQKYLETKVEPLPFASNTRVTEALKKINDWLSALGDQKTF
          	*** **********     :*  *: *:*:  ***.:      *..** *:* *.: :.:

MG06704.1 	DDARSDRSARSLGRASVKAMLNFAPALLDKMDSAIRSDDVAGLKDGLEEGKVDTSDSG--
NCU01747.1	DETKSERSTLSLGRASAKAMLNIPSALFDKLDQAIRKDEVAALEKGLKEGNLASSEGSSP
FG01473.1 	NDTMSEHSTRSLGRASAKGMLSLPQAQLDALDQAVRNDDALALEDGLKECN-AIEEG---
AN0137.1  	DDASSTHSSLSLKRGPSRPNLNLPANTLLTIDDALRKDDTHVVLELLEDLKTVADEAGEN
          	::: * :*: ** *.. :  *.:.   :  :*.*:*.*:.  : . *:: :   .:... 

MG06704.1 	-SQSLLLNLLQRSISSRSKASIAYILSQVNSLEDPDDINGRNCIHRLVLHIGRTKTTS--
NCU01747.1	LQQPLLLNLLQRSISSRSRDCIVFLLKQVNSLDEPDDINERNCIHRLVIHIGRTKTASLL
FG01473.1 	-AHPLLLGLLQRSISARSKTCISFLVSNTKTLDEPDDINSRNCIHRLVIHIGRTKAT---
AN0137.1  	VFPKVLRSLLQRAIHYRSKACISAVIGRSGSLEEEDDINKRNCIHRLVIAIGRAQTTTD-
          	    :* .****:*  **: .*  :: .  :*:: **** ********: ***:::::  

MG06704.1 	AGEQEPAPYPFPHSSKFETQMGTHYLNPATSTGHIPAA--GSESKEEKLLGHGDDAVQIL
NCU01747.1	SSEPEAESYPFPR---TGVHYAQPYLTPATSP-LSAPR--GTTLKESKLLGHDDEAIQLL
FG01473.1 	NGEQDSKSRPVP----AGSQFSNRHLEPGLTT-SKAAT--ALNQNEALLLNKDDEAVQLF
AN0137.1  	SEQSASMVLDFP-------MDTPNYITPAALPTLQPPRSFAKESDHVQHLTRSDPAVSVL
          	  :  .    .*            :: *.  .   .. : .   ..   * :.* *:.::

MG06704.1 	NYLLDSLSDDHRPGLSARDSFGRTPLHYAAQFGIVDILELIIARMQDWGQFDVSRGIDAP
NCU01747.1	KYLLENLRPDQRVALVAKDGFGRIPLHYAAQFGFVVVCQIIMRKMQDWGQFNVENGIDAP
FG01473.1 	IFLLEQLRPEQRITLKSRDSFGRMPLHYAAQFGIVVICQIIMSKMQEWGQFHVKDGIDTP
AN0137.1  	EYLLDHLRPDQRESLLSKDISGRTPLHYAAQYGFRVVCEVIIEHLQAWNMFDVSEGIDGP
          	 :**: *  ::*  * ::*  ** *******:*:  : ::*: ::* *. *.*. *** *

MG06704.1 	EWEDNEGNAPLHLSVIGGHPRTTKALLKGESWQGLSESDKTELRRNVSKSSAVLAIATKH
NCU01747.1	EWQDKDGNAPLHLSVIGGHPLTTKALLQGENWKGVNDH-QAEMRRAISKSSAVLALATKA
FG01473.1 	EWQDNDGYAPLHLSVVGGHPLTTRALLQGENWEGCSKG-KADIRKSIAKSGAVLAIATKS
AN0137.1  	RWQDNDGWAPLHLSVVGGHPLTTQALLKAENWKGQIQD-KAEIRKHVSRSSAVLALAVKA
          	.*:*::* *******:**** **:***:.*.*:*  .  ::::*: :::*.****:*.* 

MG06704.1 	NHARIVAMLANAGVDINWRDKSGETALHIAARFGHYECARILIRGTDEQKADLEVGENFF
NCU01747.1	NSKDIVELLVNAGVDINWQDNSGETALHVAARFGHDECARVLLKGTEQQKANLELAEKNF
FG01473.1 	NYSVIVQLLIEAGVDINWKDKTGETALHVAARFGHVECAQIILKGTQDQKVDLEATESTY
AN0137.1  	NFIDIVQLLVDAKVDINYQDQQGETALHVAARFDHEKCARILLKGNDVQKADTELAESTY
          	*   ** :* :* ****::*: ******:****.* :**:::::*.: **.: *  *. :

MG06704.1 	AWTPLHIAAVDGHLSVAQLLVDAGADVDKPDSSGWTAKEHAALRGHMPIARLLAAHSTVE
NCU01747.1	AWTPLHIAAVDGHLGVAQLLVDAGADVDKLDSSGWTAKEHAALRGHLDIARLLSAHSQAP
FG01473.1 	SWTPLHVAAVDGRFSVAELLVEAGADITRLDSSGWTAREHAALRGHMDIARLLETDALEA
AN0137.1  	SWTPLFIACVDGSLSVVEALIEAGADLERLDSSGWTAKEHAALRGHLDVARCLAKVTPEP
          	:****.:*.*** :.*.: *::****: : *******:********: :** *   :   

MG06704.1 	----ETATASAGESQPNQPSTGTSPPEVSSLG-----DRRSTAAVSRPAEPVRAYGHRYL
NCU01747.1	----EDEGGSSSDGDDEKSSTTTPYPETDNNTLSASLGDRRSNATSRPAEPVKTFGHRYL
FG01473.1 	----ESPPVRPVDTLQPTPSDMSSIDERRSN-------GNGANNVSRSQDAIKSFGHRYL
AN0137.1  	ELSEEPALTVPTASGSTTSGPLSSQPQSSLMEKKSNGGSAAGSSPSRNPEPVKSFGHRYL
          	. :.*     .       ..  :.  :      .:. .       **  :.::::*****

MG06704.1 	KDQTMVLVSLGTMDMRKHVDAVKLDRVPLAEAHTTQLDTALSLVLSATGAQGDSTVIDLP
NCU01747.1	KDESLVLVSLGSMDMRKGIEAIKLDKVPLAEAHTTQLDTALSVVVSAQGAKGDPTMIDLP
FG01473.1 	TNESLVLVSLGSMDMRKNVEPVSLDRVPLTEAHNTQLDTALSIVVSATGAHGEPTIIDLP
AN0137.1  	TDEAMILVSLGTMDTRKHVHAVNLDRIPMENAHATQLDTALSIVVSANGAHGEPEIIDLP
          	.:::::*****:** ** :..:.**::*: :** ********:*:** **:*:. :****

MG06704.1 	VHENICTEPVIFTTTDVTKVKLLFDIVPTYSGNEKNKIGRAVALLSSIRPSIGAKRMSLQ
NCU01747.1	VHENISTEPILFTTRDAAKVRLFFDIVPTYSGNEKNKVGRGVALLSSVRPTVGTKRMNLQ
FG01473.1 	VHDSISTEPIVFMTSEAEKVKLLFDIVPTYSGNDKNKVGRAVALLSSVKPTLGSRRMNLQ
AN0137.1  	VQENISTEPIVFHAADPTKVRLLFDLVPTYSGSKDQIVGRGVALLSSVRPSVGSHRTNLQ
          	*::.*.***::* : :  **:*:**:******...: :**.******::*::*::* .**

MG06704.1 	GDVCIPIIDSN-LDVIGAVNFNFLVITPFSHPNMEVTSKQTYWKKMSSTMVIGHRGLGKN
NCU01747.1	GDISVPIMGSN-FEVIGTVNFNFLVITPFSHPKMEVTSQQTYWKKLDSTMVIGHRGLGKN
FG01473.1 	GDVCVPIMSSN-FDVIGTVNFNFLVITPFHHPNMEVTSRQTYWKKLESTMLIGHRGLGKN
AN0137.1  	GDSTVPIVAANTLEVIGSITFNFLVITPFKHPNMSINREQTYWRSMSSTMVIGHRGLGKN
          	**  :**: :*:::***::.********* **:*.:. .****:.:.***:*********

MG06704.1 	QAANKSLQLGENTVPSFIAAANLGAQYVEFDVQLTKDHIPVIYHDFLVSETGIDAPVHTL
NCU01747.1	VVANKSLQLGENTIPSFIAAANLGAQYVEFDVQLTKDHVPVIYHDFLVSETGFDAPVHTL
FG01473.1 	LTSNKSLQLGENTLPSFIAAANLGAHYVEFDVQLTKDHVPVIYHDFLVSETGIDAPVHTL
AN0137.1  	FATRNSLQLGENTIQSFIAAANLGASYVEFDIQLTKDHVPVIYHDFLVSETGIDAPVHTL
          	 .:.:********: ********** *****:******:*************:*******

MG06704.1 	TLEQFLHINPDSSRN--------HSPNGRDSNGQPKNS----QIKKIRSNSPGPRQRSLS
NCU01747.1	TLEQFLHINPDSSRSGTHHHHHHHHHGHHGHHGQPGQQAQHRQLKKFRSNSPGPRQRSMS
FG01473.1 	TLEQFLHINSDKSRD-----SQQQAKKKPPHSGVPGD-------FRARSNSVAPK-RSQS
AN0137.1  	TLEQFLQLGERGTTR---------TPGSPGQIAIGGTE-------RSKTPPLPPRHRSMS
          	******::.   :                   .    .       : :: .  *: ** *

MG06704.1 	MGWANTDSTVLDERMKHTRDFKAKGYKANSRGNFIQAPFATLEDLFHKLPEDVGFNIEMK
NCU01747.1	MDFPGDGKDLMEERMKHTRDFKEKGYKGNSRGNFIQAPFATLEDLFRKLPESVGFNVEMK
FG01473.1 	MGFAGSGPDDLDERMKHTKDFKEQGFKGNTRGNFIQAPFATLEDLFRELPEHVGFNIEMK
AN0137.1  	VGGTESDISELNERIKHTRDFKKKGFKGNSRGNHIQAPFATLEELFKKLPQNVGFNMELK
          	:. .  .   ::**:***:*** :*:*.*:***.*********:**::**: ****:*:*

MG06704.1 	~YPMLHESEEHEMDTYAVELNSFCDTVLAKVYDLAGTRNIIFSSFNPDICLCLSFKQPNF
NCU01747.1	~YPMLHETEEHEMDTYAVELNSFCDTVLQKVYDMAGQRHIIFSSFNPDICLCLSFKQPNI
FG01473.1 	~YPMLHESEEHEMDTYAVELNSFCDTVLSKVYDLSGERHIIFSSFNPDICLCLSYKQPSI
AN0137.1  	1YPMLYESEEEEMDTYAVELNSFVDTVLEKVYTLGQGRNMIFSSFNPDICLLLSFKQPSI
          	 ****:*:**.************ **** *** :.  *::*********** **:***.:

MG06704.1 	PILFLSDAGAVPVGDVRASSLQEAIRFASRWNLLGVVSVAEPFVLSPRLVRVVKQNGLVC
NCU01747.1	PILFLTDAGTCPVGDVRASSLQEAIRFASRWNLLGIVSNAEPLINSPRLVRVVKQSGLVC
FG01473.1 	PILFLTDAGCCDVCDIRASSLQEAIRFASRWNLLGIVAAAEPFINSPRLIKIVKENGIVC
AN0137.1  	PVLFLTDSGSSPIGDIRASSLQEAIRFASRWNLLGVVTQAECLVLCPRLIRVVKESGLVC
          	*:***:*:*   : *:*******************:*: ** :: .***:::**:.*:**

MG06704.1 	VSYGVWNNDPKMVQ0RQVKEGIDAVIVDSVLAIRKGLTA---------GQEQEHSDAAQT
NCU01747.1	VSYGSQNNDPSLVQ0KQVKEGIDAVIVDSVLAIRKGLTRDQGNVNGSGGSNAKELQQRVR
FG01473.1 	FSYGVLNNDPIMVQ0RQVKQGIDAVIVDSVLAIRKGLTN---------GETPAELTNGEA
AN0137.1  	VSYGTSNNDPHKVK0LQAAEGIDAVIVDSVLAIRKGLTEHEGKNSFTPGPTPHASPLSQP
          	.***  ****  *:  *. :******************  ... . : *           

MG06704.1 	GENGEIVDLSAHPID-------------------SNNSTKSGAVVASTA----------
NCU01747.1	EVEEAINKEERIEEEGSSAEGSSYGDSNGNGNGTEIRMTTNAGGDPKTEIKETMRALRT
FG01473.1 	ETNGTLKSTHALKEK-------------------LEELTVNGAGP--------------
AN0137.1  	TINAALKDAHRIPVLN------------------NNTEVKDNYLQVKSDAASL------
          	  :  : .       .                      . .     .:   .
```
